# Hantavirus in Bat, Sierra Leone

**DOI:** 10.3201/eid1801.111026

**Published:** 2012-01

**Authors:** Sabrina Weiss, Peter T. Witkowski, Brita Auste, Kathrin Nowak, Natalie Weber, Jakob Fahr, Jean-Vivien Mombouli, Nathan D. Wolfe, Jan Felix Drexler, Christian Drosten, Boris Klempa, Fabian H. Leendertz, Detlev H. Kruger

**Affiliations:** Robert Koch-Institute, Berlin, Germany (S. Weiss, K. Nowak, F.H. Leendertz);; Charité School of Medicine, Berlin (P.T. Witkowski, B. Auste, B. Klempa, D.H. Kruger);; Ulm University, Ulm, Germany (N. Weber);; University of Braunschweig, Braunschweig, Germany (J. Fahr);; Laboratoire National de Santé Publique, Brazzaville, Republic of the Congo (J.-V. Mombouli);; Stanford University Program in Human Biology, Stanford, California, USA (N.D. Wolfe);; University of Bonn Medical Centre, Bonn, Germany (J.F. Drexler, C. Drosten);; Slovak Academy of Sciences, Bratislava, Slovakia (B. Klempa)

**Keywords:** hantavirus, rodent-borne pathogens, bats, Africa, Chiroptera, Sierra Leone, zoonosis

**To the Editor:** Hantaviruses (family *Bunyaviridae*) are transmitted from rodent reservoirs to humans. These viruses cause life-threatening human diseases: hantavirus cardiopulmonary syndrome in the Americas and hemorrhagic fever with renal syndrome in Asia and Europe (*1*). Since 2006, indigenous hantaviruses were reported also from Africa. Sangassou virus was found in an African wood mouse (*Hylomyscus simus*) in Guinea (*2*). Discovery of newer African hantaviruses, Tanganya virus and recently Azagny virus, was even more surprising because they were found in shrews (*3**,**4*).

The detection of hantaviruses in small mammals other than rodents, such as shrews and also moles (*4*), increasingly raises questions regarding the real hantavirus host range. Bats (order Chiroptera) are already known to harbor a broad variety of emerging pathogens, including other bunyaviruses (*5*). Their ability to fly and social life history enable efficient pathogen maintenance, evolution, and spread. Therefore, we conducted a study on hantaviruses in bats from Africa.

A total of 525 tissue samples from 417 bats representing 28 genera were tested for the presence of hantavirus RNA. Samples originated from different regions in western and central Africa and were collected during 2009 and early 2011. Total RNA was extracted from tissue samples and reverse transcribed. cDNA was screened by PCR specific for sequences of the large genomic segment across the genus *Hantavirus* (*2*).

One sample yielded a product of the expected size and was subjected to cloning and sequencing. The positive sample (MGB/1209) was obtained from 1 of 18 investigated slit-faced bats (family Nycteridae). The animal was trapped at the Magboi River within Gola National Park, Sierra Leone (7°50.194′N, 10°38.626′W), and the identification as *Nycteris hispida* has been verified with the voucher specimen (RCJF529). Histologic examination of organs of the animal showed no obvious pathologic findings.

The obtained 414-nt sequence covers a genomic region, which was found to correspond to nt position 2,918–3,332 in the large segment open reading frame of prototypic Hantaan virus. Bioinformatic analysis on the amino acid level showed highest degrees of identity to shrew- and mole-associated hantaviruses (Thottapalayam virus 73.0%, Altai virus 69.7%, Nova and Imjin virus 69.3%). On the basis of tree topology of a maximum-likelihood phylogenetic tree, the sequence does not cluster with rodent-associated hantaviruses but groups with those found in shrews and moles (Figure).

**Figure:**
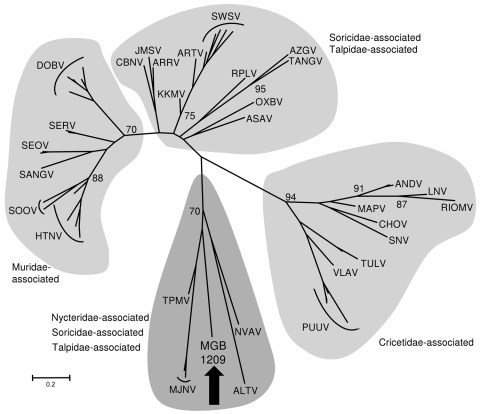
Maximum-likelihood phylogenetic tree of MGB/1209 virus based on partial large segment sequence (414 nt) and showing the phylogenetic placement of the novel sequence from *Nycteris* spp. bat compared with hantaviruses associated (i) with shrews and moles: Altai virus (ALTV), Artybash virus (ARTV), Asama virus (ASAV), Ash River virus (ARRV), Azagny virus (AZGV), Camp Ripley virus (RPLV), Cao Bang virus (CBNV), Imjin virus (MJNV), Jemez Springs virus (JMSV), Kenkeme virus (KKMV), Nova virus (NVAV), Oxbow virus (OXBV), Seewis virus (SWSV), Tanganya virus (TGNV), Thottapalayam virus (TPMV), and (ii) with rodents: Andes virus (ANDV), Choclo virus (CHOV), Dobrava-Belgrade virus (DOBV), Hantaan virus (HTNV), Laguna Negra virus (LNV), Maporal virus (MAPV), Puumala virus (PUUV), Rio Mamore virus (RIOMV), Sangassou virus (SANGV), Seoul virus (SEOV), Serang virus (SERV), Sin Nombre virus (SNV), Soochong virus (SOOV), Tula virus (TULV), Vladivostok virus (VLAV). The list of the accession numbers used in the analysis is available from the authors upon request. The tree was computed by using MEGA5 (http://www.megasoftware.net). The Tamura 3-parameter model with gamma-distributed rate heterogeneity and a proportion of invariant sites (T92 +G + I) was selected as the best fit evolutionary model according to the Baeysian information criterion calculated with MEGA5. The values at the tree branches are the bootstrap support values calculated from 500 replicates. Scale bar indicates an evolutionary distance of 0.2 substitutions per position in the sequence. The gray areas indicate association of hantaviruses with reservoir host families. The MGB/1209 partial sequence of the large genomic segment was deposited in GenBank under accession no. JN037851.

Considering that bats are more closely related to shrews and moles than to rodents (*6*), a certain genetic similarity of a putative bat-borne hantavirus with shrew- and mole-associated hantaviruses seems reasonable. Notably, shrew-associated Thottapalayam virus (India) and Imjin virus (South Korea) seem to be closer relatives, and African Tanganya virus (Guinea) and Azagny virus (Côte d’Ivoire) are more distantly related. Additional sequence data is needed for more conclusive phylogenetic analyses.

Because the new amino acid sequence is at least 22% divergent from those of other hantaviruses, we conclude that the bat was infected with a newly found hantavirus. We propose the putative name Magboi virus (MGBV) for the new virus because it was detected in an animal captured at the Magboi River in Sierra Leone. The MGBV nucleotide sequence is novel and has not been known or handled before in our laboratory. Before this study, hantavirus nucleic acid was found in lung and kidney tissues of bats from the genera *Eptesicus* and *Rhinolophus* in South Korea. However, nucleotide sequencing showed the presence of prototypical Hantaan virus indicating a spillover infection or laboratory contamination (*7*).

Further screening is necessary to confirm *N.* *hispida* as a natural reservoir host of the virus. Although the presented bat-associated sequence is obviously distinct from other hantaviruses, which suggests association with a novel natural host, a spillover infection from another, yet unrecognized host cannot be ruled out. However, detection of the virus exclusively in 1 organ (lung but not in liver, kidney, and spleen; data not shown) suggests a persistent infection that is typically observed in natural hosts of hantaviruses (*8*).

To date, only a few reports exist on cases of hemorrhagic fever with renal syndrome in Africa (*9**,**10*). However, underreporting must be assumed because the symptoms resemble those of many other febrile infections. Moreover, in cases of infections by non–rodent-associated hantaviruses, cross-reactivity with routinely used rodent-borne virus antigens should be limited and may hamper human serodiagnostics (*1*). The results suggest that bats, which are hosts of many emerging pathogens (*5*), may act as natural reservoirs for hantavirus. The effect of this virus on public health remains to be determined.

## References

[1] Krüger DH, Schonrich G, Klempa B. Human pathogenic hantaviruses and prevention of infection. Hum Vaccin. 2011;7:685–93. 10.4161/hv.7.6.15197 21508676 PMC3219076

[2] Klempa B, Fichet-Calvet E, Lecompte E, Auste B, Aniskin V, Meisel H, Hantavirus in African wood mouse, Guinea. Emerg Infect Dis. 2006;12:838–40. 16704849 10.3201/eid1205.051487 PMC3374458

[3] Klempa B, Fichet-Calvet E, Lecompte E, Auste B, Aniskin V, Meisel H, Novel hantavirus sequences in shrew, Guinea. Emerg Infect Dis. 2007;13:520–2. 10.3201/eid1303.061198 17554814 PMC2725914

[4] Kang HJ, Kadjo B, Dubey S, Jacquet F, Yanagihara R. Molecular evolution of Azagny virus, a newfound hantavirus harbored by the West African pygmy shrew (*Crocidura obscurior*) in Cote d’Ivoire. Virol J. 2011;8:373. 10.1186/1743-422X-8-373 21798050 PMC3163557

[5] Calisher CH, Childs JE, Field HE, Holmes KV, Schountz T. Bats: important reservoir hosts of emerging viruses. Clin Microbiol Rev. 2006;19:531–45. 10.1128/CMR.00017-06 16847084 PMC1539106

[6] Murphy WJ, Eizirik E, Johnson WE, Zhang YP, Ryder OA, O’Brien SJ. Molecular phylogenetics and the origins of placental mammals. Nature. 2001;409:614–8. 10.1038/35054550 11214319

[7] Jung YT, Kim GR. Genomic characterization of M and S RNA segments of hantaviruses isolated from bats. Acta Virol. 1995;39:231–3. 8825306

[8] Schönrich G, Rang A, Lutteke N, Raftery MJ, Charbonnel N, Ulrich RG. Hantavirus-induced immunity in rodent reservoirs and humans. Immunol Rev. 2008;225:163–89. 10.1111/j.1600-065X.2008.00694.x 18837782

[9] Coulaud X, Chouaib E, Georges AJ, Rollin P, Gonzalez JP. First human case of haemorrhagic fever with renal syndrome in the Central African Republic. Trans R Soc Trop Med Hyg. 1987;81:686. 10.1016/0035-9203(87)90455-X 2895515

[10] Klempa B, Koivogui L, Sylla O, Koulemou K, Auste B, Kruger DH, Serological evidence of human hantavirus infections in Guinea, West Africa. J Infect Dis. 2010;201:1031–4. 10.1086/651169 20187741

